# PET imaging in rat brain shows opposite effects of acute and chronic alcohol exposure on phosphodiesterase-4B, an indirect biomarker of cAMP activity

**DOI:** 10.1038/s41386-024-01988-y

**Published:** 2024-09-16

**Authors:** Shiyu Tang, Sung Won Kim, Amanda Olsen-Dufour, Torben Pearson, Michael Freaney, Erick Singley, Madeline Jenkins, Nathaniel J. Burkard, Aaron Wozniak, Paul Parcon, Shawn Wu, Cheryl L. Morse, Susovan Jana, Jeih-San Liow, Sami S. Zoghbi, Janaina C. M. Vendruscolo, Leandro F. Vendruscolo, Victor W. Pike, George F. Koob, Nora D. Volkow, Robert B. Innis

**Affiliations:** 1 Molecular Imaging Branch, National Institute of Mental Health, Bethesda, MD USA; 2 Laboratory of Neuroimaging, National Institute on Alcohol Abuse and Alcoholism, Bethesda, MD USA; 3 Clinical Core Laboratory, National Institute on Alcohol Abuse and Alcoholism, Bethesda, MD USA; 4 Neurobiology of Addiction Section, Integrative Neuroscience Research Branch, National Institute on Drug Abuse, Baltimore, MD USA; 5 Stress and Addiction Neuroscience Unit, Integrative Neuroscience Research Branch, National Institute on Drug Abuse Intramural Research Program, National Institute on Alcohol Abuse and Alcoholism Division of Intramural Clinical and Biological Research, Baltimore, MD USA

**Keywords:** Neuroscience, Biomarkers

## Abstract

The cyclic adenosine monophosphate (cAMP) cascade is thought to play an important role in regulating alcohol-dependent behaviors, with potentially opposite effects following acute versus chronic administration. Phosphodiesterase 4 (PDE4) is the primary brain enzyme that metabolizes cAMP, thereby terminating its signal. Radioligand binding to PDE4 serves as an indirect biomarker of cAMP activity, as cAMP-protein kinase A (PKA)-mediated phosphorylation of PDE4 increases its affinity for radioligand binding ~10-fold. Of the four PDE4 subtypes, PDE4B polymorphisms are known to be strongly associated with alcohol and substance use disorders. This study imaged rats with the PDE4B-preferring positron emission tomography (PET) radioligand [^18^F]PF-06445974 following acute and chronic ethanol administration, aiming to explore the potential of PDE4B PET imaging for future human studies. Compared to the control group treated with saline, acute alcohol administration (i.p. ethanol 0.5 g/kg) significantly increased whole brain uptake of [^18^F]PF-06445974 as early as 30 minutes post-exposure. This effect persisted at 2 hours, peaked at 4 hours, and diminished at 6 hours and 24 hours post-exposure. In contrast, in a rat model of alcohol dependence, [^18^F]PF-06445974 brain uptake was significantly reduced at 5 hours post-exposure and was normalized by 3 days. This reduction may reflect long-term adaptation to repeated alcohol-induced activation of cAMP signaling with chronic exposure. Taken together, the results suggest that PET imaging of PDE4B in individuals with alcohol use disorder (AUD) should be considered in conjunction with ongoing trials of PDE4 inhibitors to treat alcohol withdrawal and reduce alcohol consumption.

## Introduction

Alcohol use disorders (AUDs) have devastating effects on global morbidity and mortality, accounting for over 80,000 deaths annually in the United States alone. Given the heterogeneity of the disorder and the limited number of treatments, an urgent need exists for novel therapeutic strategies with unique efficacy for the various domains of AUD-related dysfunction. One promising target for novel AUD treatments is the cyclic adenosine monophosphate (cAMP) cascade, which modulates alcohol use. Studies have shown that alcohol exposure alters the cAMP pathway in vitro and in animal models. Interestingly, whereas acute alcohol exposure appears to activate cAMP signaling, chronic alcohol exposure and withdrawal reduce its activity (reviewed in [1]).

Phosphodiesterases (PDEs) are intracellular enzymes that regulate cAMP concentration by hydrolyzing cAMP and are, therefore, major targets for therapeutic intervention in the cAMP cascade. Among the 11 PDE families, PDE4 is highly expressed across the brain and has four subtypes: PDE4A-D. In the human brain, PDE4B and PDE4D are the predominant subtypes of PDE4 [2]. PDE4 is activated in the cAMP cascade through phosphorylation by protein kinase A (PKA). cAMP signaling is terminated by a negative feedback loop in which PKA, activated by cAMP, activates PDE4, which then degrades cAMP through hydrolysis. Because PDE4 activation is a consequence of cAMP activation, the level of activated PDE4 can be used as a biomarker for cAMP signaling activity.

Previous rodent studies found that stimulating the cAMP pathway with PDE4 inhibitors reduced alcohol consumption, alcohol preference, and alcohol withdrawal symptoms [3, 4]. Furthermore, two PDE4-selective or PDE4-preferred inhibitors—apremilast and ibudilast—have shown preliminary evidence of effectiveness in reducing alcohol consumption in individuals with AUD [5, 6], highlighting the therapeutic potential of targeting PDE4 in the treatment of AUD.

Current PDE4 inhibitors, known as pan-PDE4 inhibitors, such as rolipram, inhibit all PDE4 subtypes but often induce varying degrees of emetic side effects that significantly limit their therapeutic potential. These adverse effects are associated with PDE4D inhibition in the brainstem’s emetic center [7], underscoring the need for effective treatments with an improved therapeutic profile. PDE4B is the main subtype associated with mood regulation and neuroinflammation, two key aspects of AUD. It is the predominant subtype in brain regions critical to reward and affect, including the hypothalamus, ventral striatum, globus pallidus, and substantia nigra [8]. PDE4B inhibition has been linked to antidepressant-like effects, an outcome not observed with PDE4D inhibition [9]. PDE4B is also the primary subtype in microglia and macrophages. Its expression can be induced by lipopolysaccharide (LPS), and PDE4B ablation has been shown to attenuate LPS-induced inflammatory processes [10, 11]. Given that alcohol exposure triggers neuroinflammation, PDE4B may play a role in alcohol-induced neuroinflammation. Furthermore, genetic variants in PDE4B have been associated with substance use disorders, including alcohol use disorder [12, 13]. The possible association between PDE4B brain levels and alcohol exposure has been studied in ex vivo rodent models. For example, one study found that chronic binge-like drinking increased *Pde4b* gene expression in alcohol-preferring mice [5]. In another study, week-long exposure to alcohol increased *Pde4b* gene expression and protein levels while reducing brain cAMP levels [14]. Collectively, these findings identify PDE4B as a promising target to help counteract the pathophysiology of AUD with a better therapeutic profile than pan-PDE4 inhibitors.

Previous positron emission tomography (PET) imaging studies of PDE4 revealed that cAMP-PKA-mediated phosphorylation increased the affinity of radiolabeled molecule (‘radioligand’) binding [15]. PET imaging using [^11^C](*R*)-rolipram detected significant increases and decreases in binding following modulation of the cAMP pathway via PKA activators and inhibitors, respectively [15], confirming the association between [^11^C](*R*)-rolipram binding and cAMP activity. In individuals with McCune-Albright Syndrome (MAS), a mosaic disorder characterized by gain-of-function mutations in the alpha subunit of the stimulatory G protein (G_αs_) and adenylate cyclase (AC) activity, increased [^11^C](*R*)-rolipram binding was noted in areas of dysplastic bone compared with healthy volunteers [16]. Overall, these findings support using [^11^C](*R*)-rolipram to evaluate PDE4 phosphorylation by PKA and, consequently, cAMP activity in vivo.

The novel PDE4B-preferring radioligand [^18^F]PF-06445974 was recently evaluated in rodents, monkeys, and humans [17]. This radioligand demonstrates widespread distribution in the brain and can quantify PDE4B in the human brain [18]. Building on this work, this study sought to use PET imaging with [^18^F]PF-06445974 to assess the effects of both acute and chronic alcohol exposure on PDE4B radioligand binding in rat brains in vivo. The hypothesis was that alcohol exposure would modulate brain cAMP activity, thereby altering PDE4B activity, a process observable through changes in [^18^F]PF-06445974 binding. The study serves as a translational bridge for PDE4B PET studies in individuals with AUD. The findings will help to elucidate the potential of PET imaging of PDE4B as an indirect biomarker for monitoring the cAMP cascade in vivo in humans during alcohol use and misuse.

## Materials and Methods

### Animals

The experiments were conducted in accordance with the Guide for Care and Use of Laboratory Animals [19] and were approved by the Animal Care and Use Committee of the National Institute on Drug Abuse (NIDA) and National Institute of Mental Health (NIMH).

Adult male Wistar rats were used for both the acute and chronic alcohol exposure experiments. Acute alcohol exposure was achieved with intraperitoneal injection (IP) of ethanol (0.5 g/kg, 20% v/v in saline). Chronic alcohol exposure was induced using the intermittent alcohol vapor model that produces somatic and motivational signs of withdrawal, as previously described [20–22]. Briefly, alcohol-dependent (DEP) rats were exposed to alcohol vapor for 14 hours (i.e., intoxication) followed by 10 hours of room air (i.e., withdrawal). This exposure cycle occurred daily for at least six weeks. The non-dependent (NON-DEP) control rats were exposed to room air during the same period.

### PET imaging and data analysis

For rats in the acute alcohol exposure group and their saline controls (*n* = 3 for each timepoint, weight: 304±31 g), PET scans were performed at 0.5, 2, 4, 6, and 24 hours following acute alcohol or saline administration. For rats in the chronic alcohol exposure group and their control counterparts, PET imaging occurred during the withdrawal period. Ten DEP (598±85 g) and 8 NON-DEP (632±75 g) rats were scanned at approximately 5 hours after termination of chronic alcohol exposure. A separate group of 9 DEP and 7 NON-DEP rats were scanned 3 days after termination of chronic alcohol exposure (DEP: 512±57 g; NON-DEP: 532±71 g) and again between 2 to 3 weeks post-exposure (hereafter referred to as 2 weeks; DEP: 510±51 g; NON-DEP: 510±59 g).

To determine the effect of efflux transporters on between-group differences, an additional 2 DEP and 2 NON-DEP rats were injected with the efflux transporter (P-glycoprotein [P-gp] and breast cancer resistance protein [BCRP]) inhibitor elacridar (3 mg/kg) at 50 minutes during the PET scan that took place at 5 hours after termination of chronic alcohol exposure. Elacridar (Sigma-Aldrich Co., St. Louis, MO, USA) was dissolved in *N,N*-dimethylacetamide/propylene glycol/PEG-400/isotonic saline (10/10/30/50 by volume) in concentrations of up to 1.5 mg/mL. The volume of injection was up to 2 ml/kg.

Preparation of [^18^F]PF-06445974 and PET scans using the LFER 150 PET-CT (Mediso Ltd., Budapest, Hungary) were performed as previously described [23, 24]. Briefly, rats were anesthetized with about 4% isoflurane in oxygen, and anesthesia was maintained with 1-3% isoflurane during PET scanning. Heart rate and oxygen saturation values were monitored throughout the scan to guide the adjustment of anesthesia level. CT scans for attenuation correction and image analysis were acquired before radioligand injection. [^18^F]PF-06445974 was administered through an intravenous catheter (BTPE-10, Instech Laboratories, Plymouth Meeting, PA, USA) as a bolus at 0.5 mL/min. Brain dynamic scans started immediately at radioligand injection and were acquired for 120 minutes. PET images were reconstructed using the 3D ordered subsets expectation maximization algorithm.

Image analysis was performed with PMOD 4.302 (PMOD Technologies; Zurich, Switzerland). PET images were co-registered to the W. Schiffer rat brain atlas in PMOD using CT image as the reference image [25]. Time activity curves were extracted from the whole brain and brain regions critical to alcohol exposure (i.e., nucleus accumbens and striatum). Standardized uptake value (SUV) was obtained to compensate for variations in body weight and injected activity. The area under the time activity curve (AUC) was calculated to reflect brain uptake of the radioligand.

### Blood and brain sample processing

Radiometabolite-corrected arterial plasma input functions (AIFs) were obtained from an additional two pairs of rats 0.5 hours after acute exposure and two pairs 5 hours after chronic exposure. Eleven anticoagulated femoral artery blood samples (0.2–0.5 mL each, with the last being 0.5 mL) were collected at intervals ranging from 0.5 to 120 minutes post-radioligand injection. After removing whole blood aliquots (0.01 mL each), plasma was separated by centrifugation. Both whole blood and plasma aliquots (0.01 mL each) were quantified for radioactivity using an automatic γ-counter (model 2480 Wizard 2; Perkin-Elmer, Waltham, MA, USA), decay-corrected to the injection time.

The parent radioligand fraction was determined by deproteinating plasma samples (0.08 mL each, with the last being 0.1 mL) through acetonitrile treatment (0.3 mL). After centrifugation, clear supernates were analyzed using reversed-phase radio-high-performance liquid chromatography (radio-HPLC) on an X-terra C_18_ column. Elution was done with isocratic methanol:water:triethylamine (65:35:0.1; v/v) at 5.0 mL/min, as previously detailed [26]. The injected volumes were 0.08 mL of plasma (or a total volume of 0.38 mL of acetonitrile and plasma; the last sample was 0.4 mL). Eluates were monitored with an in-line NaI_*(Tl)*_ scintillation detector, and data were analyzed using “Bio-ChromeLite”. Data for each radiochromatogram were decay-corrected according to their respective HPLC injection times.

At 120 minutes post-radioligand injection, rats were euthanized via thoracotomy and exsanguination, and brain tissues were extracted. Brains were homogenized with twice their weights in acetonitrile, followed by 1.0 mL of water. After centrifugation, clear supernates were injected onto the HPLC column through nylon filters for analysis of radioactive compositions, as previously described [26].

### Measurement of plasma-free fraction

The plasma free fraction (*f*_p_) for [^18^F]PF-06445974 was measured in 4 DEP and 4 NON-DEP rats 5 hours into withdrawal from alcohol exposure using ultrafiltration through membrane filters (Centrifree; Millipore; Burlington, MA), as previously described [27]; these rats were separate from those used in PET imaging. A control *f*_p_ value was measured from a human pooled standard plasma in each run to control for inter-assay variability [28]. The *f*_p_ value from each rat plasma sample was corrected based on the ratio between the control *f*_p_ of the corresponding run and the averaged control *f*_p_ across all runs.

### Gas chromatography analysis of blood and brain after acute exposure

The concentration of ethanol in rat blood (*n* = 3) and brain (*n* = 1) samples was measured using headspace gas chromatography with flame ionization detection, as described elsewhere [29]. Prior to ethanol injection, a catheter (BTPU-027, Instech Laboratories) was inserted into the femoral artery. Arterial blood samples (100 uL) were collected at 0, 10, 20, 30, 40, 50, 60, and 120 minutes after IP ethanol administration (0.5 g/kg, 20% in saline). Each rat brain was harvested immediately after the final blood sampling and was homogenized in saline. An aliquot of each sample (20 µL) was mixed with an internal standard solution (400 µL, 0.42 mM 1-propanol in saline). Each sample was analyzed with the gas chromatography system (7890A, Agilent Technologies, Santa Clara, CA, USA) equipped with a headspace autosampler (HT2000H, HTA s.r.l) and a capillary column (J&W DB-ALC2, 30 m x 0.32 mm × 1.20 µm, Agilent Technologies).

### Western blot

Brain tissues were extracted from four pairs of rats (4 NON-DEP and 4 DEP), including two pairs immediately following the PET scan 5 hours into withdrawal (pairs 1 & 2) and another two pairs at a similar time point post-alcohol exposure (pairs 3 & 4). The left hemispheres were homogenized in RIPA lysis butter (ThermoFisher, Waltham, MA) and centrifuged at 8,000 x *g* for 10 minutes at 4 °C. The protein concentration of the supernatant was determined using NanoDrop. An aliquot of 20 μg protein from the supernatant was combined 1:1 ratio with 2X Laemmli sample buffer containing β-mercaptoethanol and incubated at 95 °C for 5 minutes. These samples were then electrophoresed on a 4–20% Mini-PROTEAN TGX Precast cell (Bio-Rad, San Francisco, CA) and transferred onto PVDF membrane using the Bio-Rad Trans-Blot Turbo transfer system (Bio-Rad, San Francisco, CA). The membrane was blocked with the Scanlater blocking buffer (Molecular Devices, LLC, San Jose, California) for 1 hour at room temperature before being incubated with a primary antibody cocktail: Rabbit anti-PDE4B2 (ABS181, MilliporeSigma, 1:1000) and Mouse anti-Beta-actin (ab8226, Abcam, 1:2500). The membrane was subsequently incubated with europium-labeled secondary antibody cocktail: ScanLater Eu-Anti-Mouse IgG and ScanLater Eu-Anti-Rabbit IgG (Molecular Devices, 1:1000 each).

The signals were detected using Molecular Devices SpectraMax Imager. The generated image was quantitatively analyzed for protein levels using ImageJ v1.53k (NIH, USA). PDE4B concentration was normalized with the concentration of β-actin. Duplicates were obtained for the second set of two pairs of rats (pairs 3 & 4), and the averaged ratios across duplicates were calculated as the final value for each rat for between group comparison.

### Statistical analysis

Between-group comparisons of brain radioactivity following acute alcohol exposure and at 5 hours following chronic alcohol exposure were performed with two-sample Student’s *t*-tests. Because the PET scan (except for two DEP rats) and the *f*_p_ measurement at 5 hours following chronic alcohol exposure were performed in pairs (e.g., one NON-DEP and one DEP rat at a time), paired two-sample Student’s *t*-tests were performed to control for inter-experiment variability. Given that the same rats were scanned at 3 days and 2 weeks following termination of chronic exposure, a repeated measures ANOVA was performed in GraphPad Prism (Version 5.02 for Windows, GraphPad Software, Boston, Massachusetts USA, www.graphpad.com) to assess the effect of alcohol by controlling for the effect of time and the interaction between time and alcohol exposure.

## Results

### Effects of acute alcohol exposure

**Fig. 1:**
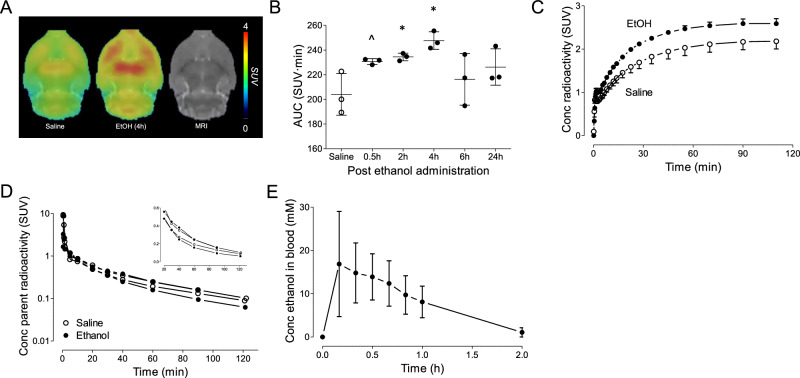
Brain and plasma levels of [^18^F]PF-06445974 and blood alcohol levels after acute alcohol exposure. **A** Group-averaged standardized uptake value (SUV) images (averaged from 50 to 100 minutes) of saline and alcohol-exposed rats at 4 hours post-exposure. **B** The area under the time-activity curve (AUC) of the whole brain of saline- and alcohol-exposed rats at multiple time points after exposure. Whole brain radioactivity increased at 0.5 hours following acute alcohol exposure, peaked at 4 hours, and normalized by 6 hours. **C** A representative example of group-averaged whole brain time-activity curves at 4 hours post-saline or alcohol exposure. **D** Plasma level of parent radioactivity of two pairs of saline and alcohol exposed rats at 0.5 hours post-exposure; inserted window shows the later portion in linear scale. **E** Blood alcohol levels following alcohol administration in a group of three naïve rats. ^*p* < 0.06, *p < 0.05 difference to the saline group. EtOH: acute alcohol-exposed group.

Widespread increases in brain [^18^F]PF-06445974 levels were observed in rats following acute alcohol administration; an example of brain SUV images is shown in Fig. 1A. Compared to the saline control group (AUC: 204.0 ± 16.9), acute alcohol administration increased whole brain uptake of [^18^F]PF-06445974 as early as 0.5 hours post-exposure (230.7 ± 2.3, *p* = 0.054). This effect persisted at 2 hours (234.4 ± 3.0, *p* = 0.038), peaked at 4 hours (247.7 ± 7.1, *p* = 0.015), and diminished at 6 hours (216.2 ± 20.8, *p* = 0.476) and 24 hours (226.2 ± 14.7, *p* = 0.162) (Fig. 1B). [^18^F]PF-06445974 uptake in nucleus accumbens and striatum followed a similar pattern to that observed in the whole brain, increasing at 2 hours after alcohol administration and returning to normal levels 24 hours post-administration (Supplementary Fig. S1). [^18^F]PF-06445974 whole brain uptake for the 4-hour post-acute exposure was consistently higher than that of the saline group throughout the scan time; a representative example is shown in Fig. 1C. Plasma levels of [^18^F]PF-06445974 at 0.5 hours post-exposure were comparable between the acute alcohol- and saline-treated rats (Fig. 1D). In both groups, radiometabolites were not detected in brain samples (data not shown). Blood alcohol levels following administration peaked before 0.5 hours (13.9 ± 5.3 mM) and were almost cleared from the system by approximately 2 hours (Fig. 1E), suggesting that changes in brain PDE4B radioligand binding were likely a delayed or secondary effect of alcohol exposure rather than a direct consequence of its presence in the bloodstream. The mean peak blood alcohol concentration was ~ 17 mM, which translates to around 80 mg%. This is the approximate blood alcohol level achieved following binge drinking in humans [30] and in human studies following ethanol IV administration [31]. No observable acetaldehyde was detected in the brain or blood, and the blood-to-brain ratio was 0.71 (*n* = 1) at 0.5 hours after acute alcohol administration (data not shown).

### Effects of chronic alcohol exposure

**Fig. 2:**
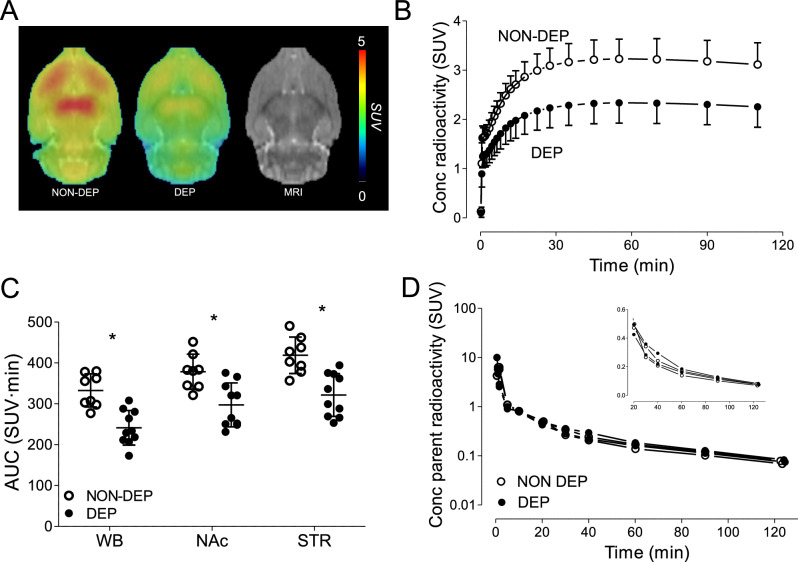
Brain and plasma levels of [^18^F]PF-06445974 in rats at 5 hours following chronic alcohol exposure. Brain radioactivity decreased without altering the level of plasma parent. **A** Group-averaged standardized uptake value (SUV) images (averaged from 50 to 100 minutes). **B** Group-averaged whole brain time-activity curves of NON-DEP and DEP rats. **C** The area under the time-activity curve (AUC) of the whole brain, nucleus accumbens, and striatum. **D** Plasma level of parent radioactivity in two pairs of NON-DEP and DEP rats; inserted window shows the later portion in linear scale. *p < 0.001 between group differences. NON-DEP: non-dependent; DEP: dependent; WB: whole brain; NAc: nucleus accumbens; STR: striatum.

**Fig. 3:**
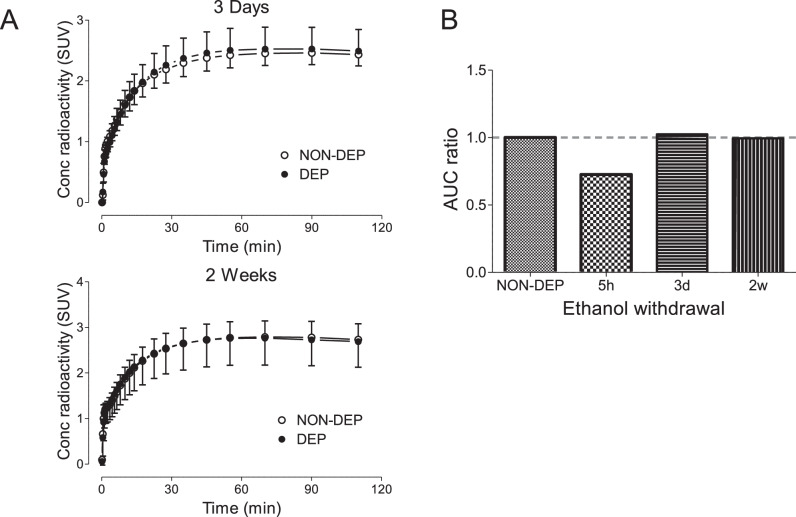
Brain uptake of [^18^F]PF-06445974 in rats at 3 days and 2 weeks following chronic alcohol exposure. **A** Group-averaged whole brain time-activity curves of NON-DEP and DEP rats at 3 days (top) and 2 weeks (bottom) post exposure. **B** The ratio of area under the time-activity curve (AUC) between DEP and NON-DEP groups at each time point. NON-DEP non-dependent, DEP dependent.

Whole brain [^18^F]PF-06445974 levels were reduced in DEP rats compared to NON-DEP rats at approximately 5 hours after chronic exposure (Fig. 2A, B). This NON-DEP versus DEP difference (AUC values) was widespread across the whole brain (332 ± 41 versus 241 ± 42, *p* < 0.001) and was significant in nucleus accumbens (378 ± 43 versus 297 ± 54, *p* = 0.003) and striatum (418 ± 45 versus 321 ± 52, *p* < 0.001), as assessed via two-sample *t*-tests (Fig. 2C). Because rats were scanned in pairs (except for two DEP rats), reduced [^18^F]PF-06445974 levels were validated using paired two-sample *t*-tests on eight pairs of rats to account for potential confounding variables across study days. The paired two-sample *t*-tests confirmed the significant difference between NON-DEP versus DEP rats in the whole brain (332 ± 41 versus 247 ± 39, *p* = 0.009), nucleus accumbens (378 ± 43 versus 284 ± 51, *p* = 0.014), and striatum (418 ± 45 versus 313 ± 55, *p* = 0.010). Plasma levels of [^18^F]PF-06445974 at 5 hours post-exposure were similar between NON-DEP and DEP rats (Fig. 2D), and the *f*_p_ of [^18^F]PF-06445974 showed no significant difference between NON-DEP (8.61% ± 0.98%) and DEP (7.11% ± 0.64%, *p* = 0.061) rats based on paired two-sample *t*-tests. Radiometabolites were not detected in either NON-DEP or DEP brains (data not shown). The observed reduction of brain [^18^F]PF-06445974 levels was not present at later time points (that is, at 3 days and 2 weeks following termination of chronic exposure) (Fig. 3). The repeated measures ANOVA detected no significant main effect of alcohol treatment (*p* = 0.910) or a significant interaction between alcohol treatment and time (*p* = 0.733), although a significant main effect of time was observed (*p* = 0.009), likely caused by general experimental conditions other than alcohol exposure.

**Fig. 4:**
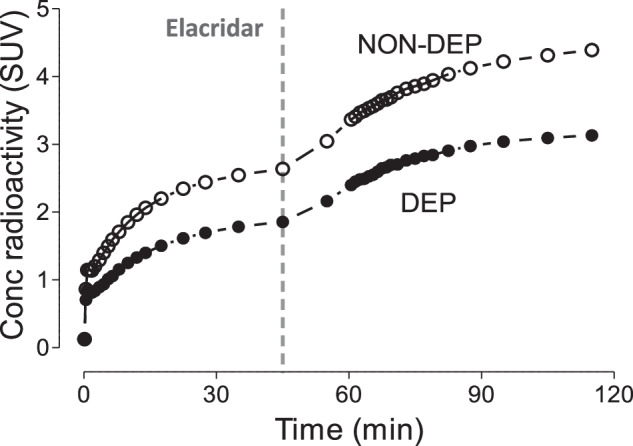
Brain uptake of [^18^F]PF-06445974 in rats at 5 hours following chronic alcohol exposure pre- and post-efflux transporter inhibition by elacridar. NON-DEP non-dependent, DEP dependent.

A previous study from our laboratory found that [^18^F]PF-06445974 is a substrate for key brain efflux transporters (P-gp and BCRP) [17]. To determine whether the reduced [^18^F]PF-06445974 uptake at 5 hours post-exposure merely reflected increased removal from the brain, efflux transporters were inhibited with the dual-efflux inhibitor elacridar in an additional two pairs of rats. As expected, injection of elacridar 50 minutes into the PET scan increased brain levels of [^18^F]PF-06445974 in both NON-DEP and DEP rats (Fig. 4). However, inhibition of the efflux transporters did not alter the observed differences between groups; the averaged brain SUV was 29.8% lower in DEP rats pre-inhibition (averaged from 30 to 50 minutes) and 28.5% lower post-inhibition (averaged from 100 to 120 minutes).

**Fig. 5:**
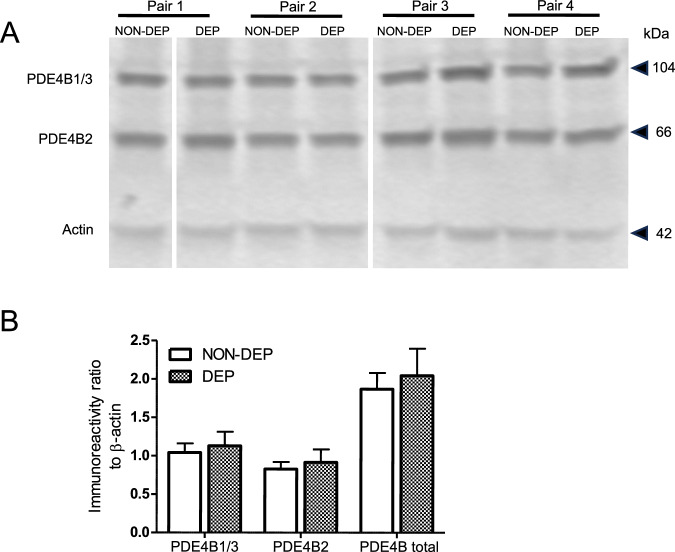
Western blot analysis of rat brain tissues following chronic alcohol exposure. **A** Western blot showing the PDE4B1/3, PDE4B2, and actin immunoreactivity from four pairs of rats. One set from the duplicate is shown for pair 3 and pair 4. **B** Averaged immunoreactivity relative to actin of NON-DEP and DEP brain tissues. NON-DEP non-dependent, DEP dependent.

The Western blot analysis revealed no significant differences in PDE4B protein levels between NON-DEP and DEP brains (Fig. 5, full blot available in Supplementary Fig. S2). The mean concentration ratios of PDE4B2 and PDE4B1/3 relative to β-actin were comparable between NON-DEP and DEP brains, determined by a paired two-sample Student’s *t*-test (PDE4B2: *p* = 0.490; PDE4B1/3: *p* = 0.556).

## Discussion

This study found that acute alcohol exposure rapidly increased PDE4B radioligand binding in rat brains as early as 0.5 hours post-exposure, an effect that peaked at 4 hours and returned to normal by 6 hours. In contrast, chronic alcohol exposure decreased PDE4B radioligand binding 5 hours into the withdrawal, an effect that had normalized by 3 days and 2 weeks post-exposure. These opposing changes in PDE4B radioligand binding align with theories of altered cAMP activity reported in previous in vitro and ex vivo studies, as discussed below. In addition, the radiometabolite-corrected AIF remained unaffected by either acute or chronic alcohol exposure. The observed reduction of PDE4B radioligand binding following chronic alcohol exposure persisted after inhibition of brain efflux transporters. Taken together, these findings provide in vivo evidence that both acute and chronic alcohol exposure alter brain cAMP pathway dynamically depending on the exposure condition. Notably, the effects of alcohol on PDE4B radioligand binding occurred independently of peripheral factors and efflux transporter function.

PDE4 is known to be activated in the cAMP cascade through PKA phosphorylation, which increases the affinity of rolipram and [^11^C](*R*)-rolipram binding in PET imaging [15, 32, 33]. In this context, [^11^C](*R*)-rolipram binding serves as an indicator of cAMP activity. A similar study is being planned to confirm the correlation between cAMP activity and [^18^F]PF-06445974 binding. Although the specific characteristics of [^18^F]PF-06445974 compared to [^11^C](*R*)-rolipram remain to be fully understood, our observations are consistent with previously reported alcohol-induced changes in cAMP activity (reviewed in [1]) and suggest that alterations in [^18^F]PF-06445974 binding better reflect changes in PKA phosphorylation than total PDE4B protein levels post-alcohol exposure. Notably, alterations in [^18^F]PF-06445974 binding were observed as soon as 0.5 hours post-exposure, a timeframe that may be too brief for the upregulation of protein synthesis. For chronic alcohol exposure, the Western blot indicated no change in total PDE4B protein levels during the early withdrawal phase, further supporting the above interpretation of PET imaging observed in the present study.

Previous studies found that acute alcohol exposure activates cAMP signaling. In vitro studies detected increased cellular cAMP levels and cAMP-response element (CRE)-mediated gene expression following acute ethanol treatment [34, 35]. Similar upregulation in the cAMP cascade was also observed ex vivo in rodent brains post-ethanol injection, characterized by elevated cAMP levels, PKA activity, CRE-binding protein (CREB) phosphorylation, and CRE-mediated gene expression [36, 37]. Conversely, chronic alcohol exposure downregulates cAMP signaling, possibly as an adaptative response to prolonged alcohol use. For instance, in neuroblastoma-glioma hybrid cells, acute alcohol exposure increased adenosine receptor-stimulated cAMP levels, and these levels decreased following chronic exposure in the absence of alcohol [38]. A similar trend of reduced cAMP signaling, including reduced AC activity and CREB phosphorylation, was also detected in rodents during chronic alcohol exposure and withdrawal [39–43]. cAMP signaling and CREB both play a unique role in neurogenesis and synaptic plasticity and have been implicated in the pathology of mental disorders such as depression and anxiety [44–47]. The reduced cAMP signaling following chronic alcohol exposure may relate to anxiety- and depressive-like behaviors observed during alcohol withdrawal [48, 49], which may in turn lead to repeated alcohol misuse and relapse. In the present study, the direction of change in PDE4B radioligand binding mirrored alterations in cAMP activity following both acute and chronic alcohol exposure. This suggests that PET imaging of PDE4B could serve as an indirect in vivo biomarker of cAMP turnover in alcohol-associated conditions, warranting future application in human studies. Clinical studies have demonstrated the efficacy of PDE4-selective (apremilast) or PDE4-preferred (ibudilast) inhibitors in treating individuals with AUD [5, 6]. However, questions remain regarding whether apremilast effectively crosses the blood-brain barrier in humans and animals. This issue is particularly critical because it impacts our understanding of the therapeutic mechanisms of apremilast. PET imaging of PDE4B could provide a method to address this question directly in humans.

[^18^F]PF-06445974 is a substrate of the brain efflux transporters P-gp and BCRP [17], which raises the possibility that alcohol exposure could confound [^18^F]PF-06445974 brain uptake by affecting these transporters. However, ethanol itself is not a substrate for these efflux transporters [50], and acute alcohol exposure does not alter the function of P-gp [51]. Therefore, our finding of changes in brain uptake of [^18^F]PF-06445974 as early as 0.5 hours following acute alcohol exposure are unlikely to be attributable to altered efflux transporter activity. Nevertheless, the impact of prolonged alcohol exposure on the levels of these transporters is less clear, and previous studies yielded mixed results [52, 53]. It should be noted that the present study did not primarily seek to assess the effect of alcohol on efflux transporters. Rather, it sought to determine whether altered [^18^F]PF-06445974 uptake was confounded by these transporters. Administration of an efflux transporter inhibitor did not affect the observed between-group differences in [^18^F]PF-06445974 uptake, suggesting that the effects of chronic alcohol exposure occurred independently of potential changes in efflux transporters. Nevertheless, whether chronic alcohol exposure regulates these transporters remains an open question for future studies specifically designed to explore their relationship.

One limitation of the present study is the absence of direct measurement of PKA-phosphorylation on PDE4B. PDE4B exists as different splice variants, with PDE4B1/3 being the long forms that include a PKA-phosphorylation site in contrast to the short form PDE4B2 that lacks this site and is not directly regulated by the cAMP-PKA system. This study found that alcohol exposure did not alter the total protein levels of either PDE4B1/3 or PDEB2. Thus, it can be inferred that the observed changes in radioligand binding are due to modifications in binding affinity, likely stemming from altered PKA-phosphorylation that reflects cAMP activity. However, future research should seek to provide direct evidence of PKA phosphorylation changes and investigate the potential involvement of other pathways. For example, in contrast to the long forms of PDE4 that contain the PKA phosphorylation site, short forms of PDE4, such as PDE4B2, are activated by phosphorylation through extracellular signal-related kinases (ERKs). Increased expression of PDE4B or PDE4B2 has been reported in neuroinflammatory conditions, including prolonged alcohol exposure [5, 14, 54]. Therefore, changes in [^18^F]PF-06445974 brain uptake could also reflect ERK-mediated activation of PDE4B’s short form, which is not regulated by cAMP activity. This alternative mechanism should be considered and investigated in future studies.

In summary, our findings revealed distinct patterns of PDE4B radioligand binding in response to acute and chronic alcohol exposure, consistent with the known ability of cAMP to increase PDE4 activity and the affinity of PDE4 radioligands. In addition, decreased PDE4B radioligand binding after chronic ethanol administration in the present study suggests decreased cAMP activity, which is consistent with current research efforts to develop treatments for withdrawal by inhibiting PDE4 to increase cAMP activity. Collectively, these results highlight the potential of PDE4B PET imaging to serve as an indirect biomarker of cAMP. While the underlying mechanisms require future investigation, this study lays a promising foundation for translation to humans with AUD. Due to the high cost and time-intensive nature of PET studies, future research with PDE4B PET should focus on human AUD volunteers rather than mechanistic exploration in animals. Investigating whether similar patterns can be detected in humans could provide valuable insights on the role of the cAMP cascade in AUD and its association with withdrawal symptoms as well as its usefulness for exploring the potential therapeutic value of PDE4B inhibitors.

## Supplementary information

Supplementary material

## Data Availability

The PET images from the present study are available from the corresponding author upon request.
