# Supplementary material for: PET imaging in rat brain shows opposite effects of acute and chronic alcohol exposure on phosphodiesterase-4B, an indirect biomarker of cAMP activity
Source: Neuropsychopharmacology. 2024 Sep 16;50(2):444–51. doi: 10.1038/s41386-024-01988-y (PMC11632093; doi:10.1038/s41386-024-01988-y)
Supplement: Supplementary file 1 — Supplementary material [file 41386_2024_1988_MOESM1_ESM.docx]

*Tang et al — PET Imaging in Rat Brain Shows Opposite Effects of Acute and Chronic Alcohol Exposure on Phosphodiesterase-4B, an Indirect Biomarker of cAMP Activity*

**Supplementary Material**


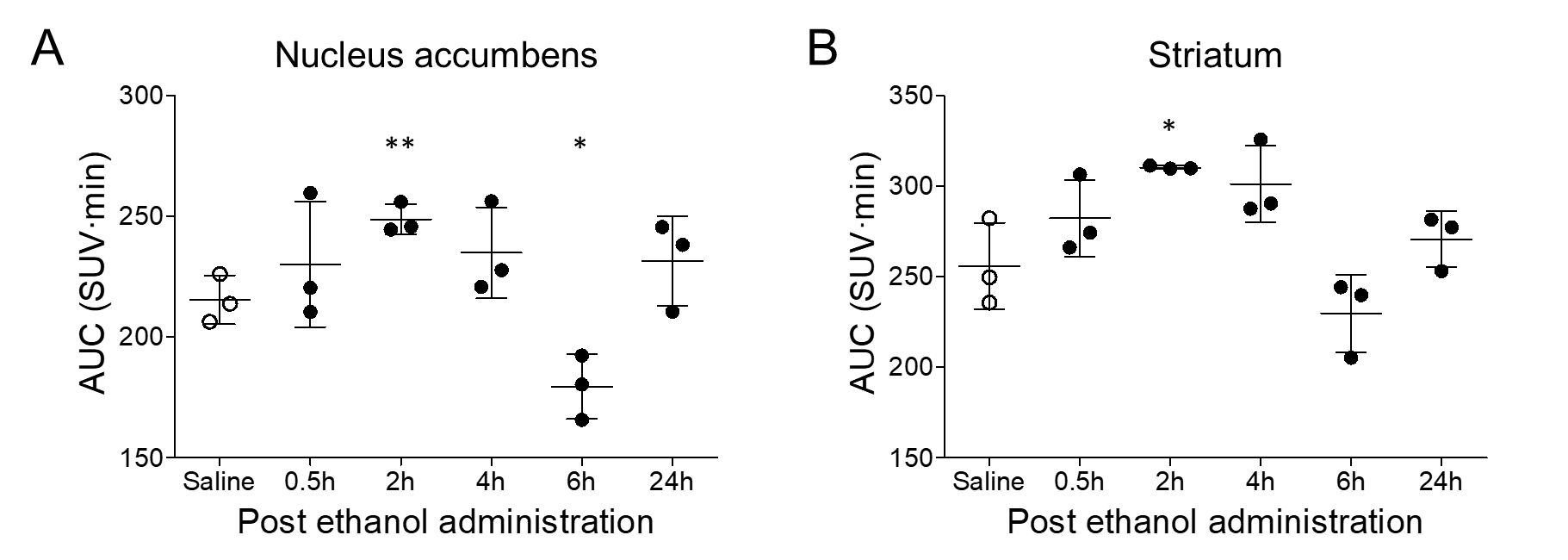


**Supplementary Figure S1**. The area under the time-activity curve (AUC) of the nucleus accumbens (A) and striatum (B) of saline- and alcohol-exposed rats at multiple time points after exposure. *p<0.05, **p<0.01 difference to the saline group.


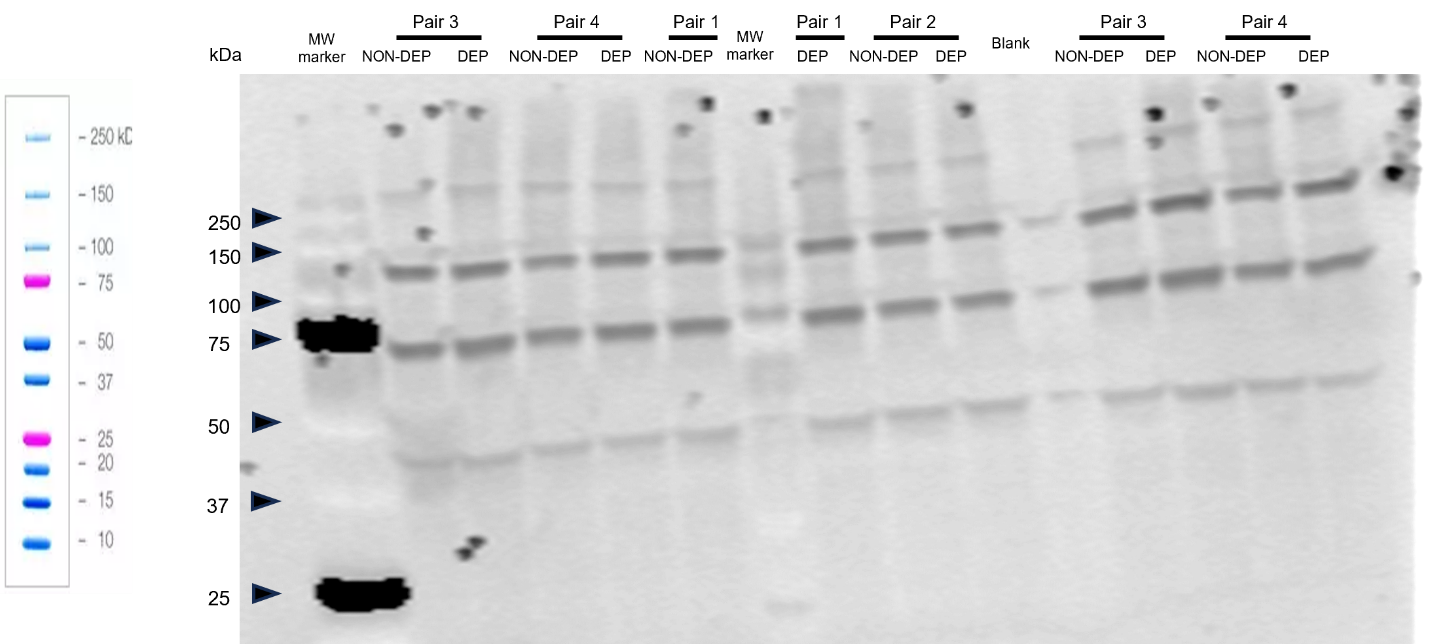


**Supplementary Figure S2**. Western blot analysis of rat brain tissues following chronic alcohol exposure. NON-DEP: non-dependent; DEP: dependent.
